# Supplementary material for: Heparin-based hydrogel scaffolding alters the transcriptomic profile and increases the chemoresistance of MDA-MB-231 triple-negative breast cancer cells
Source: Biomater Sci. 2020 Feb 13;8(10):2786–96. doi: 10.1039/c9bm01481k (PMC7497406; doi:10.1039/c9bm01481k)
Supplement: Supplementary file 2 [file BM-008-C9BM01481K-s002.zip › Supplementary File 4/HP-BhydrogelvControl/Pathways/my_analysis.Gsea.1545201079907/HALLMARK_ESTROGEN_RESPONSE_EARLY.html]

Details for gene set HALLMARK\_ESTROGEN\_RESPONSE\_EARLY[GSEA]

|  || Dataset | expr.class.cls#HYD\_versus\_CONTROL.class.cls#HYD\_versus\_CONTROL\_repos |
| Phenotype | class.cls#HYD\_versus\_CONTROL\_repos |
| Upregulated in class | HYD |
| GeneSet | HALLMARK\_ESTROGEN\_RESPONSE\_EARLY |
| Enrichment Score (ES) | 0.18044196 |
| Normalized Enrichment Score (NES) | 0.7348963 |
| Nominal p-value | 0.96638656 |
| FDR q-value | 1.0 |
| FWER p-Value | 1.0 |
Table: GSEA Results Summary

  

Fig 1: Enrichment plot: HALLMARK\_ESTROGEN\_RESPONSE\_EARLY      
 Profile of the Running ES Score & Positions of GeneSet Members on the Rank Ordered List

  

| PROBE | DESCRIPTION (from dataset) | GENE SYMBOL | GENE\_TITLE | RANK IN GENE LIST | RANK METRIC SCORE | RUNNING ES | CORE ENRICHMENT || 1 | STC2 | na |  |  | 313 | 2.647 | 0.0058 | Yes |
| 2 | LRIG1 | na |  |  | 449 | 2.415 | 0.0190 | Yes |
| 3 | FASN | na |  |  | 563 | 2.266 | 0.0321 | Yes |
| 4 | WWC1 | na |  |  | 569 | 2.254 | 0.0508 | Yes |
| 5 | CELSR1 | na |  |  | 625 | 2.178 | 0.0662 | Yes |
| 6 | SLC22A5 | na |  |  | 826 | 2.011 | 0.0726 | Yes |
| 7 | MLPH | na |  |  | 876 | 1.974 | 0.0866 | Yes |
| 8 | NAV2 | na |  |  | 906 | 1.942 | 0.1014 | Yes |
| 9 | THSD4 | na |  |  | 1140 | 1.773 | 0.1040 | Yes |
| 10 | RAPGEFL1 | na |  |  | 1274 | 1.703 | 0.1114 | Yes |
| 11 | ESRP2 | na |  |  | 1468 | 1.596 | 0.1146 | Yes |
| 12 | NCOR2 | na |  |  | 1743 | 1.474 | 0.1126 | Yes |
| 13 | ADCY9 | na |  |  | 1794 | 1.444 | 0.1221 | Yes |
| 14 | RRP12 | na |  |  | 2114 | 1.308 | 0.1164 | Yes |
| 15 | SH3BP5 | na |  |  | 2155 | 1.295 | 0.1251 | Yes |
| 16 | NADSYN1 | na |  |  | 2276 | 1.248 | 0.1293 | Yes |
| 17 | BAG1 | na |  |  | 2573 | 1.152 | 0.1234 | Yes |
| 18 | KDM4B | na |  |  | 2687 | 1.122 | 0.1269 | Yes |
| 19 | SCARB1 | na |  |  | 2693 | 1.119 | 0.1361 | Yes |
| 20 | MYBBP1A | na |  |  | 2787 | 1.089 | 0.1403 | Yes |
| 21 | MED13L | na |  |  | 2805 | 1.084 | 0.1486 | Yes |
| 22 | SYNGR1 | na |  |  | 2882 | 1.067 | 0.1535 | Yes |
| 23 | MED24 | na |  |  | 3080 | 1.017 | 0.1517 | Yes |
| 24 | HES1 | na |  |  | 3253 | 0.976 | 0.1509 | Yes |
| 25 | TTC39A | na |  |  | 3351 | 0.952 | 0.1538 | Yes |
| 26 | CYP26B1 | na |  |  | 3501 | 0.917 | 0.1537 | Yes |
| 27 | FOXC1 | na |  |  | 3523 | 0.912 | 0.1602 | Yes |
| 28 | AKAP1 | na |  |  | 3529 | 0.910 | 0.1676 | Yes |
| 29 | ITPK1 | na |  |  | 3751 | 0.858 | 0.1632 | Yes |
| 30 | MYC | na |  |  | 3771 | 0.854 | 0.1694 | Yes |
| 31 | SEMA3B | na |  |  | 3816 | 0.847 | 0.1742 | Yes |
| 32 | RASGRP1 | na |  |  | 3920 | 0.828 | 0.1757 | Yes |
| 33 | UNC119 | na |  |  | 4035 | 0.805 | 0.1765 | Yes |
| 34 | DLC1 | na |  |  | 4227 | 0.765 | 0.1729 | Yes |
| 35 | MREG | na |  |  | 4240 | 0.764 | 0.1787 | Yes |
| 36 | KAZN | na |  |  | 4366 | 0.739 | 0.1783 | Yes |
| 37 | CELSR2 | na |  |  | 4443 | 0.725 | 0.1804 | Yes |
| 38 | TPBG | na |  |  | 4814 | 0.656 | 0.1665 | No |
| 39 | SLC37A1 | na |  |  | 4893 | 0.643 | 0.1678 | No |
| 40 | TBC1D30 | na |  |  | 5149 | 0.606 | 0.1595 | No |
| 41 | FAM102A | na |  |  | 5184 | 0.602 | 0.1628 | No |
| 42 | SLC7A5 | na |  |  | 5187 | 0.602 | 0.1677 | No |
| 43 | FARP1 | na |  |  | 5271 | 0.589 | 0.1683 | No |
| 44 | TIPARP | na |  |  | 5438 | 0.563 | 0.1643 | No |
| 45 | SYBU | na |  |  | 5522 | 0.549 | 0.1646 | No |
| 46 | EGR3 | na |  |  | 6013 | 0.483 | 0.1429 | No |
| 47 | TPD52L1 | na |  |  | 6047 | 0.480 | 0.1452 | No |
| 48 | ALDH3B1 | na |  |  | 6050 | 0.480 | 0.1491 | No |
| 49 | TFAP2C | na |  |  | 6126 | 0.467 | 0.1491 | No |
| 50 | FOS | na |  |  | 6167 | 0.461 | 0.1509 | No |
| 51 | MAPT | na |  |  | 6363 | 0.435 | 0.1443 | No |
| 52 | IGF1R | na |  |  | 6443 | 0.424 | 0.1437 | No |
| 53 | ISG20L2 | na |  |  | 6490 | 0.418 | 0.1448 | No |
| 54 | IL17RB | na |  |  | 6523 | 0.413 | 0.1466 | No |
| 55 | SYT12 | na |  |  | 6552 | 0.409 | 0.1485 | No |
| 56 | INHBB | na |  |  | 6568 | 0.407 | 0.1512 | No |
| 57 | TJP3 | na |  |  | 6670 | 0.396 | 0.1492 | No |
| 58 | PODXL | na |  |  | 6709 | 0.391 | 0.1505 | No |
| 59 | SLC1A4 | na |  |  | 6805 | 0.382 | 0.1487 | No |
| 60 | SLC1A1 | na |  |  | 6810 | 0.382 | 0.1517 | No |
| 61 | SLC9A3R1 | na |  |  | 6845 | 0.378 | 0.1531 | No |
| 62 | ASB13 | na |  |  | 6846 | 0.378 | 0.1563 | No |
| 63 | AMFR | na |  |  | 6900 | 0.371 | 0.1566 | No |
| 64 | SVIL | na |  |  | 7043 | 0.354 | 0.1521 | No |
| 65 | REEP1 | na |  |  | 7044 | 0.354 | 0.1551 | No |
| 66 | PDZK1 | na |  |  | 7086 | 0.350 | 0.1559 | No |
| 67 | FHL2 | na |  |  | 7132 | 0.343 | 0.1564 | No |
| 68 | KLF4 | na |  |  | 7185 | 0.341 | 0.1565 | No |
| 69 | CCND1 | na |  |  | 7494 | 0.308 | 0.1429 | No |
| 70 | AFF1 | na |  |  | 7495 | 0.308 | 0.1455 | No |
| 71 | ABCA3 | na |  |  | 7496 | 0.308 | 0.1481 | No |
| 72 | JAK2 | na |  |  | 7561 | 0.298 | 0.1473 | No |
| 73 | BCL2 | na |  |  | 7623 | 0.289 | 0.1465 | No |
| 74 | MICB | na |  |  | 7630 | 0.288 | 0.1486 | No |
| 75 | OLFML3 | na |  |  | 7650 | 0.285 | 0.1500 | No |
| 76 | RPS6KA2 | na |  |  | 7768 | 0.277 | 0.1462 | No |
| 77 | ANXA9 | na |  |  | 7794 | 0.273 | 0.1472 | No |
| 78 | DHRS2 | na |  |  | 7852 | 0.267 | 0.1464 | No |
| 79 | GAB2 | na |  |  | 7901 | 0.261 | 0.1461 | No |
| 80 | CHPT1 | na |  |  | 8205 | 0.231 | 0.1321 | No |
| 81 | RARA | na |  |  | 8244 | 0.227 | 0.1320 | No |
| 82 | TMEM164 | na |  |  | 8276 | 0.222 | 0.1322 | No |
| 83 | WFS1 | na |  |  | 8337 | 0.215 | 0.1309 | No |
| 84 | RHOBTB3 | na |  |  | 8872 | 0.156 | 0.1041 | No |
| 85 | CANT1 | na |  |  | 9012 | 0.153 | 0.0981 | No |
| 86 | PPIF | na |  |  | 9104 | 0.140 | 0.0945 | No |
| 87 | AQP3 | na |  |  | 9407 | 0.104 | 0.0795 | No |
| 88 | XBP1 | na |  |  | 9718 | 0.073 | 0.0638 | No |
| 89 | DHRS3 | na |  |  | 9773 | 0.068 | 0.0616 | No |
| 90 | MAST4 | na |  |  | 9787 | 0.066 | 0.0614 | No |
| 91 | ABAT | na |  |  | 9813 | 0.062 | 0.0606 | No |
| 92 | ABLIM1 | na |  |  | 9912 | 0.052 | 0.0559 | No |
| 93 | FKBP5 | na |  |  | 9936 | 0.048 | 0.0551 | No |
| 94 | DHCR7 | na |  |  | 10032 | 0.036 | 0.0504 | No |
| 95 | MYB | na |  |  | 10213 | 0.015 | 0.0411 | No |
| 96 | RAB17 | na |  |  | 10501 | -0.005 | 0.0261 | No |
| 97 | SCNN1A | na |  |  | 10551 | -0.010 | 0.0236 | No |
| 98 | UGCG | na |  |  | 10616 | -0.018 | 0.0204 | No |
| 99 | MYBL1 | na |  |  | 10630 | -0.020 | 0.0199 | No |
| 100 | TMPRSS3 | na |  |  | 10856 | -0.046 | 0.0084 | No |
| 101 | PDLIM3 | na |  |  | 10867 | -0.049 | 0.0083 | No |
| 102 | DEPTOR | na |  |  | 10903 | -0.054 | 0.0069 | No |
| 103 | KRT15 | na |  |  | 11062 | -0.073 | -0.0008 | No |
| 104 | CD44 | na |  |  | 11081 | -0.075 | -0.0011 | No |
| 105 | FKBP4 | na |  |  | 11149 | -0.086 | -0.0039 | No |
| 106 | ELF3 | na |  |  | 11330 | -0.093 | -0.0126 | No |
| 107 | TSKU | na |  |  | 11335 | -0.094 | -0.0120 | No |
| 108 | KLF10 | na |  |  | 11366 | -0.099 | -0.0127 | No |
| 109 | SIAH2 | na |  |  | 11609 | -0.127 | -0.0244 | No |
| 110 | PTGES | na |  |  | 11658 | -0.133 | -0.0258 | No |
| 111 | ZNF185 | na |  |  | 11711 | -0.139 | -0.0273 | No |
| 112 | SNX24 | na |  |  | 11720 | -0.141 | -0.0266 | No |
| 113 | MYOF | na |  |  | 11838 | -0.156 | -0.0314 | No |
| 114 | NXT1 | na |  |  | 11839 | -0.156 | -0.0301 | No |
| 115 | CLIC3 | na |  |  | 11878 | -0.160 | -0.0308 | No |
| 116 | SEC14L2 | na |  |  | 11885 | -0.161 | -0.0297 | No |
| 117 | SFN | na |  |  | 12185 | -0.198 | -0.0438 | No |
| 118 | TUBB2B | na |  |  | 12229 | -0.204 | -0.0443 | No |
| 119 | TGIF2 | na |  |  | 12335 | -0.218 | -0.0480 | No |
| 120 | RBBP8 | na |  |  | 12693 | -0.242 | -0.0647 | No |
| 121 | KRT8 | na |  |  | 12716 | -0.245 | -0.0638 | No |
| 122 | NBL1 | na |  |  | 13075 | -0.295 | -0.0802 | No |
| 123 | FRK | na |  |  | 13207 | -0.312 | -0.0844 | No |
| 124 | B4GALT1 | na |  |  | 13415 | -0.340 | -0.0924 | No |
| 125 | RHOD | na |  |  | 13484 | -0.351 | -0.0931 | No |
| 126 | BHLHE40 | na |  |  | 13518 | -0.356 | -0.0918 | No |
| 127 | P2RY2 | na |  |  | 13598 | -0.368 | -0.0929 | No |
| 128 | SLC7A2 | na |  |  | 13664 | -0.379 | -0.0931 | No |
| 129 | TGM2 | na |  |  | 13799 | -0.403 | -0.0968 | No |
| 130 | ADCY1 | na |  |  | 13936 | -0.415 | -0.1004 | No |
| 131 | ABHD2 | na |  |  | 14131 | -0.431 | -0.1070 | No |
| 132 | SLC19A2 | na |  |  | 14203 | -0.441 | -0.1070 | No |
| 133 | FDFT1 | na |  |  | 14374 | -0.474 | -0.1120 | No |
| 134 | TIAM1 | na |  |  | 14991 | -0.561 | -0.1396 | No |
| 135 | WISP2 | na |  |  | 15043 | -0.569 | -0.1375 | No |
| 136 | SLC2A1 | na |  |  | 15114 | -0.583 | -0.1363 | No |
| 137 | PEX11A | na |  |  | 15218 | -0.600 | -0.1367 | No |
| 138 | ARL3 | na |  |  | 15415 | -0.636 | -0.1416 | No |
| 139 | BLVRB | na |  |  | 15546 | -0.661 | -0.1429 | No |
| 140 | CLDN7 | na |  |  | 15583 | -0.666 | -0.1392 | No |
| 141 | CISH | na |  |  | 15622 | -0.672 | -0.1355 | No |
| 142 | PAPSS2 | na |  |  | 15945 | -0.732 | -0.1463 | No |
| 143 | INPP5F | na |  |  | 16031 | -0.754 | -0.1444 | No |
| 144 | CA12 | na |  |  | 16047 | -0.759 | -0.1389 | No |
| 145 | BCL11B | na |  |  | 16175 | -0.783 | -0.1389 | No |
| 146 | OPN3 | na |  |  | 16178 | -0.783 | -0.1325 | No |
| 147 | KRT18 | na |  |  | 16203 | -0.788 | -0.1271 | No |
| 148 | PMAIP1 | na |  |  | 16591 | -0.869 | -0.1401 | No |
| 149 | GLA | na |  |  | 16858 | -0.944 | -0.1462 | No |
| 150 | KRT19 | na |  |  | 16930 | -0.965 | -0.1418 | No |
| 151 | SLC26A2 | na |  |  | 17094 | -1.013 | -0.1419 | No |
| 152 | ADD3 | na |  |  | 17325 | -1.093 | -0.1448 | No |
| 153 | TFF1 | na |  |  | 17389 | -1.117 | -0.1387 | No |
| 154 | HR | na |  |  | 17401 | -1.118 | -0.1299 | No |
| 155 | RAB31 | na |  |  | 17417 | -1.120 | -0.1212 | No |
| 156 | ENDOD1 | na |  |  | 17504 | -1.156 | -0.1161 | No |
| 157 | PLA2G16 | na |  |  | 17571 | -1.185 | -0.1096 | No |
| 158 | AR | na |  |  | 17770 | -1.274 | -0.1093 | No |
| 159 | SLC39A6 | na |  |  | 17790 | -1.282 | -0.0995 | No |
| 160 | ELF1 | na |  |  | 17853 | -1.311 | -0.0917 | No |
| 161 | CALB2 | na |  |  | 17941 | -1.342 | -0.0850 | No |
| 162 | TOB1 | na |  |  | 17952 | -1.348 | -0.0742 | No |
| 163 | AREG | na |  |  | 18122 | -1.456 | -0.0709 | No |
| 164 | NRIP1 | na |  |  | 18335 | -1.591 | -0.0686 | No |
| 165 | ELOVL5 | na |  |  | 18504 | -1.707 | -0.0631 | No |
| 166 | IL6ST | na |  |  | 18809 | -2.032 | -0.0620 | No |
| 167 | PRSS23 | na |  |  | 18840 | -2.072 | -0.0462 | No |
| 168 | GJA1 | na |  |  | 18902 | -2.173 | -0.0311 | No |
| 169 | DYNLT3 | na |  |  | 19045 | -2.502 | -0.0175 | No |
| 170 | HSPB8 | na |  |  | 19137 | -3.038 | 0.0032 | No |
Table: GSEA details [plain text format]

  

Fig 2: HALLMARK\_ESTROGEN\_RESPONSE\_EARLY      
 Blue-Pink O' Gram in the Space of the Analyzed GeneSet

  

Fig 3: HALLMARK\_ESTROGEN\_RESPONSE\_EARLY: Random ES distribution      
 Gene set null distribution of ES for **HALLMARK\_ESTROGEN\_RESPONSE\_EARLY**
